# The selective pressures on the microbial community in a metal-contaminated aquifer

**DOI:** 10.1038/s41396-018-0328-1

**Published:** 2018-12-06

**Authors:** Hans K. Carlson, Morgan N. Price, Mark Callaghan, Alex Aaring, Romy Chakraborty, Hualan Liu, Jennifer V. Kuehl, Adam P. Arkin, Adam M. Deutschbauer

**Affiliations:** 1 Environmental Genomics and Systems Biology Division, Lawrence Berkeley National Laboratory, Berkeley, CA 94720 USA; 2 Earth and Environmental Sciences Area, Lawrence Berkeley National Laboratory, Berkeley, CA 94720 USA; 3 Department of Bioengineering, University of California, Berkeley, CA 94720 USA; 4 Department of Plant and Microbial Biology, University of California, Berkeley, CA 94720 USA

**Keywords:** Microbial ecology, Environmental chemistry

## Abstract

In many environments, toxic compounds restrict which microorganisms persist. However, in complex mixtures of inhibitory compounds, it is challenging to determine which specific compounds cause changes in abundance and prevent some microorganisms from growing. We focused on a contaminated aquifer in Oak Ridge, Tennessee, USA that has large gradients of pH and widely varying concentrations of uranium, nitrate, and many other inorganic ions. In the most contaminated wells, the microbial community is enriched in the *Rhodanobacter* genus. *Rhodanobacter* abundance is positively correlated with low pH and high concentrations of uranium and 13 other ions and we sought to determine which of these ions are selective pressures that favor the growth of *Rhodanobacter* over other taxa. Of these ions, low pH and high UO_2_^2+^, Mn^2+^, Al^3+^, Cd^2+^, Zn^2+^, Co^2+^, and Ni^2+^ are both (a) selectively inhibitory of a *Pseudomonas* isolate from an uncontaminated well vs. a *Rhodanobacter* isolate from a contaminated well, and (b) reach toxic concentrations (for the *Pseudomonas* isolate) in the *Rhodanobacter*-dominated wells. We used mixtures of ions to simulate the groundwater conditions in the most contaminated wells and verified that few isolates aside from *Rhodanobacter* can tolerate these eight ions. These results clarify which ions are likely causal factors that impact the microbial community at this field site and are not merely correlated with taxonomic shifts. Furthermore, our general high-throughput approach can be applied to other environments, isolates, and conditions to systematically help identify selective pressures on microbial communities.

## Introduction

Microorganisms survive within an *n*-dimensional biogeochemical space comprised of diverse nutrients and stressors [1]. While neutral effects such as drift, dispersal, and speciation influence microbial communities, selective pressures are the biogeochemical determinants that drive changes in microbial community composition based on variations in the relative fitness of microbial sub-populations [2]. Often selective pressures are inferred based on correlations between the relative abundances of microbial taxa and geochemical parameters (e.g., toxic stressors or nutrients), but correlative analysis does not identify causal relationships, and observed correlations can be misleading. Thus, laboratory approaches to measure microbial fitness in biogeochemical gradients can be very useful to help identify likely selective pressures. Given the complexity of microbial ecosystems, high-throughput approaches are essential to evaluate the relative fitness of microbial sub-populations in multi-dimensional biogeochemical gradients [3].

In both pristine and anthropogenically perturbed environments, toxic inorganic ions can impact the growth, survival, and activity of microbial sub-populations and thereby alter the composition of microbial communities [4–6]. Many soils and aquifer matrices have naturally high levels of various toxic elements, and, depending on the prevailing geochemical conditions, these elements can be solubilized as free inorganic ions and impact biological systems [5, 7]. For example, in the rhizosphere, inorganic ions are solubilized from soil minerals by organic acids released from plant roots. In this acidic and high metal environment, acid- and metal-tolerant taxa are favored [8, 9]. Some contaminated environments vary even more in pH and inorganic ion concentration, and taxonomic shifts are correlated with changes in ion concentrations in wastewater treatment plants [10], urban estuaries [5], acid mine drainage [11, 12], and metal-contaminated aquifers [13–16]. Studies into the microbial ecology of these sites is important for understanding and mitigating the impact of ion toxicity on ecosystem health [4], but they also represent useful sites for testing general principles of microbial ecology.

The aquifer at the US Department of Energy Field Research Center in Oak Ridge, Tennessee (ORFRC) has large gradients of pH and inorganic ions [17]. At the ORFRC, between 1951 and 1983, nitric acid-solubilized uranium (U) waste from the Y-12 nuclear processing plant was deposited in the unlined S-3 ponds along with mixed metal and organic wastes from other DOE (Department of Energy) facilities [17]. The intrusion of low pH waste further dissolved the fractured shale and karst aquifer matrix releasing other inorganic ions. For example, dissolution of clay minerals releases Mn^2+^, Al^3+^, and other transition metals [18, 19], while dissolution of carbonate minerals releases alkali earth metal cations such as Ca^2+^ and Mg^2+^ [18]. Consequently, the aquifer’s pH ranges from 3 to 11 and the concentration range of many inorganic ions in groundwater is over five orders of magnitude [16, 20]. Identifying which toxic inorganic ions impact the microbial community at this field site is important for interpreting microbial community datasets [14, 16], for understanding the selective pressures that influence observed metal resistance phenotypes [21–24] and for designing optimum bioremediation strategies [25].

A number of studies describe microbial taxonomic shifts associated with geochemical extremes at the ORFRC [14, 16, 26, 27]. For example, *Rhodanobacter* often dominate the microbial community in the most contaminated wells with groundwater pH below 4, nitrate concentrations above 5 mM, and uranium concentrations above 2.5 μM, and *Rhodanobacter* is considered an indicator for contamination at the site [14, 16, 22, 26]. *Rhodanobacter* isolates from the ORFRC, and other environments, can tolerate low pH and possess genes predicted to be involved in metal tolerance [26, 28, 29]. Thus, toxic inorganic ions are dominant factors that influence microbial community composition at this site [16], but it is unknown precisely which ions are selective pressures that favor *Rhodanobacter* over other taxa.

In this study, using a combination of laboratory and field data, we tested the hypothesis that *Rhodanobacter* at the ORFRC are selectively enriched in the most contaminated wells because they are resistant to a subset of ions. Leveraging geochemical and microbial community data from a field survey [16], we identified 15 inorganic ions that are correlated with elevated relative abundance of *Rhodanobacter*. Next, we used high-throughput cultivation to identify eight of these that are both (a) selectively toxic to sensitive microbial isolates from uncontaminated wells at the ORFRC vs. resistant *Rhodanobacter* from the most contaminated wells, and (b) reach toxic concentrations likely to limit the growth of sensitive isolates, but not *Rhodanobacter*. To further validate our results, we confirmed the toxicity of mixtures of the selective ions simulating conditions in the most contaminated wells against 194 bacterial isolates from the site. Our results clarify which ions are the likely dominant selective pressures at this contaminated site, and not merely correlated with taxonomic shifts. Our general approach can be applied to other environments to help identify causal factors that influence the relative fitness of microbial sub-populations.

## Materials and methods

### Comparison of relative abundance data for *Pseudomonas* and *Rhodanobacter* with field concentrations of inorganic ions

Field concentrations of inorganic ions and 16S ribosomal DNA (rDNA)-sequencing data (Table S1) for different wells at the ORFRC were obtained from the Supplementary dataset and through personal communication with the authors of the previous field survey publication [16] (Table S1). Spearman's rank correlations between pairs of field parameters and the eight microbial genera with highest relative abundance were calculated in R version 3.4.3 (Table S2). We tested whether each pair of ions (excluding self pairs) was significantly correlated, and *p* values were converted to false discovery rates with the p.adjust method. Separately, we tested whether each ion was correlated with any of the top 8 genera and converted those *p* values to false discovery rates.

### Inorganic ion arrays

We prepared aqueous solutions of 80 inorganic ions in 96-well format to capture a wide range of elements from the periodic table in a variety of redox states and to test the influence of chelation on toxicity (Table S3). Solutions were prepared at concentrations close to the solubility limit or at concentrations that we expected, and based on previous studies, would likely be inhibitory to many ion-sensitive bacterial isolates from the ORFRC [23, 30]. Nitrilotriacetic acid (NTA) complexes were prepared to test the influence of chelation on the toxicity of some transition metals by mixing equimolar inorganic ion and the chelator, NTA. We used sodium and chloride salts as counter-ions whenever possible so that sodium chloride could serve as a control for salt stress. All chemicals are from Sigma-Aldrich (St. Louis, MO, USA).

While the oxidation state of most ions was not measured in the field survey, multiple oxidation states are represented in our inorganic ion arrays. Our ion arrays contain multiple oxidation states for an element when those oxidation states are soluble and stable in aerobic, neutral pH aqueous stock solutions. Insoluble compounds are less likely to impact microorganisms. Thus, unless we report multiple oxidation states, we posit that the inorganic ion salt in our array is in the dominant soluble oxidation state of the element measured in ORFRC groundwater samples.

Sterile stock solutions were prepared using 50 mL Steriflip filter units (EMD Millipore, Hayward, CA, USA), transferred to 15 mL conical tubes (BD Biosciences, San Jose, CA, USA), and then a Freedom Evo liquid handling robot (Tecan Group Ltd., M**ä**nnendorf, Switzerland) was used to transfer 1 mL of stock solution into deep-well 96- well plates (Costar, Thermo Fisher Scientific, Waltham, MA, USA) in the arrayed layout reported in Table S3. For growth assays, we used a Biomek FxP (Beckman Coulter, Indianapolis, IN, USA) liquid handling robot to serially dilute stock solutions into three 384-well flat-bottom transparent microplates (Costar, Thermo Fisher Scientific). Within each plate each compound is present at four concentrations. Control compounds were added in a checkerboard pattern in columns A, B, G, and H. Positive controls were a high concentration of the antibiotic chloramphenicol (0.2 g/L), which prevented growth of all microbial isolates. Negative controls were water. Each well in the 384-well assay plates contained a final volume of 40 μL aqueous solution. Stock and assay plates were sealed with foil seals (Thermo Fisher Scientific), stored at −80 °C, and thawed 48 h prior to inoculation. For anaerobic cultures, assay plates were kept unsealed in an anaerobic chamber (COY, Grass Lake, MI, USA) for 48 h prior to inoculation.

### Media and cultivation conditions

*Pseudomonas fluorescens* FW300-N2E2 (N2E2) was isolated from groundwater from background wells up-gradient from the contaminated area at the ORFRC, and previous studies suggest that it is relatively sensitive to many metals [23]. *Rhodanobacter* sp. FW104-10B01 (10B01) was isolated from a well within the contaminant plume by direct plating on R2A (R2A, HiMedia, Mumbai, India) agar plates and aerobic growth at 30 °C. *Pseudomonas fluorescens* FW300-N2E2 was recovered from −80 °C freezer stocks in LB (Luria-Bertani broth, Thermo Fisher Scientific) aerobically. *Rhodanobacter* sp. FW104-10B01 was recovered from −80 °C freezer stocks in R2A media aerobically. Overnight cultures were washed three times with chemically defined media lacking vitamins, minerals, and carbon sources before resuspension in media for dose–response assays. Basal chemically defined medium and KB medium recipes are provided in Table S4. For anaerobic, nitrate-reducing growth assays, 10 mM nitrate was added to LB and stored in an anaerobic chamber for 1 week prior to inoculation. N2E2 does not grow fermentatively under anaerobic conditions in LB. For pH profiles, R2A was amended with 30 mM HOMOPIPES (homopiperazine-N,N′-bis(2-ethanesulfonic acid), pH 3, pH 4), MES (2-(*N*-morpholino)ethanesulfonic acid, pH 5, pH 6), or PIPES (piperazine-*N*,*N*′-bis(2-ethanesulfonic acid), pH 7) (buffer salts from Sigma-Aldrich). For all dose–response screens, microbial cells were added to assay plates in an anaerobic chamber (Coy) with a 10% CO_2_:5% H_2_:85% N_2_ atmosphere using a Liquidator 96 (Mettler-Toledo, Oakland, CA, USA). Forty microliters of microbial cultures at an optical density (OD 600) of 0.04 in 2× media was added to 384-well flat bottom transparent assay microplates (Costar) containing 40 μL of aqueous compound stocks to obtain a final OD 600 of 0.02, and a final volume of 80 μL. Aerobic assay microplates were sealed with BreathEasy seals (E&K Scientific, Santa Clara, CA, USA) and grown in a Multitron (Infors, Bottmingen, Switzerland) shaker/incubator at 700rpm at 30 °C. Anaerobic assay microplates were sealed with transparent plate seals (Thermo Fisher Scientific) and grown in the anaerobic chamber without shaking. Growth was monitored by measuring OD 600 with a Tecan M1000 Pro microplate reader (Tecan Group Ltd, Männendorf, Switzerland) using the iControl software package and exported to a Microsoft Excel spreadsheet.

### Dose–response analysis

Microplate absorbance reads were uploaded to an in-house growth curve database using custom scripts. A 24-h timepoint was chosen for most dose–response analysis as this timepoint represents early stationary phase for N2E2 in the media conditions tested. 10B01 grows more slowly in R2A than does N2E2 and therefore we measured the relative inhibition based on growth at 48 h for comparisons between N2E2 and 10B01, but, for N2E2, similar relative inhibition was observed at 24 and 48 h. All growth assays were carried out in duplicate or triplicate and were repeated on different days with similar results. Positive controls (0.2 g/L chloramphenicol, 0% growth) and negative controls (water, 100% growth) were used to determine % inhibition values relative to controls. Data analysis for dose–response inhibition experiments was carried out using the drc R package [31, 32] and relative inhibition was plotted vs. the concentration of the compound and fitted to a standard inhibition dose–response curve to generate a half maximal inhibitory concentration (IC_50_) value. In some cases, compound precipitation in growth media impacted absorbance reads. To account for these cases, we removed microplate wells with initial absorbance reads greater than 1.2-fold above control wells from the IC_50_ calculation. Ninety-five percent confidence intervals for IC_50_s are reported and all IC_50_s are calculated from at least two biological replicate dose–response curves. For compounds that did not inhibit, or always inhibited >50% across the range of concentrations we screened, we indicate the highest concentration or lowest concentration screened in the Supplementary Dataset, Table S5. Comparisons between growth conditions to assess compounds with differential inhibitory potency are for cultures inoculated on the same day. We define compounds to be selective if the ratio between the IC_50_s (selectivity index, SI) is greater than 2-fold and the 95% confidence intervals do not overlap. For uranium and pH, dose–response data was analyzed using GraphPad Prism 7 (GraphPad Software Inc., La Jolla, CA, USA) and analysis of variance was used to compare fits between growth conditions to confirm that the differences between fits for selective compounds were statistically significant. In support of this approach, for replicate dose–response experiments with rich media aerobic cultures of N2E2 (LB broth, LB/aerobic), all compounds display similar inhibitory potencies with overlapping 95% confidence intervals (Figure S1). IC_50_s with confidence intervals are reported in Table S5.

### Arrayed isolate collection

Aside from N2E2 and 10B01, 192 other isolates were obtained from groundwater samples from the ORFRC using a variety of aerobic and anaerobic enrichment strategies (Table S6), but all isolates are capable of growth in R2A or LB media aerobically. The arrayed culture collection was prepared from single colony picks and grown in LB or R2A overnight before cryo-preservation in 25% glycerol. Strains were classified using 16S rDNA Sanger sequencing and the RDP (Ribosomal Database Project) classifier [33] and are indicated in Table S6.

Arrayed isolates were recovered in R2A in 96 deep-well blocks (Costar) aerobically with shaking at 700 rpm in a Multitron plate shaker/incubator (Infors). Overnight cultures were centrifuged to pellet cells. Cells were resuspended in buffered R2A media in the presence of inorganic ions at the mean concentrations observed in wells with >5% *Rhodanobacter* at an OD 600 between 0.01 and 0.06 for sensitivity assays. As mentioned above, 30 mM pH buffers were used for pH profiles. In control experiments, N2E2 IC_50_s were not affected by the addition of 30 mM PIPES to R2A. An isolate was scored as inhibited in a given condition if growth was at least 2 standard deviations below the average of four control no-stress cultures.

To identify abundant sequence types in 16S data from the site [16], we downloaded the reads from MG-RAST (https://www.mg-rast.org/linkin.cgi?project=mgp8190). We used usearch (https://www.drive5.com/usearch/) to filter out reads with more than one expected error; we extracted the region between the primers (the V4 region) with a custom perl script; and we used usearch to count the abundance of each sequence type in each sample.

## Results

### Identification of inorganic ion parameters correlated with high relative abundance of *Rhodanobacter* at the ORFRC

**Fig. 1:**
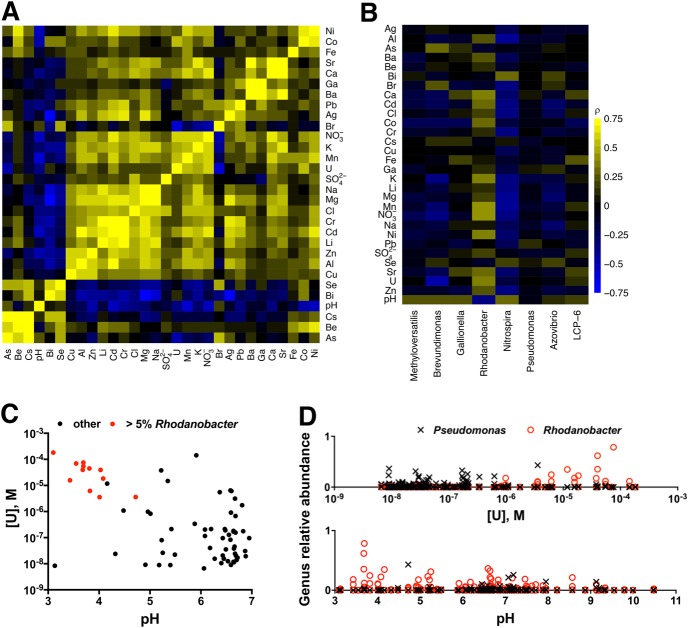
Correlations between concentrations of ions and the dominant genera at the ORFRC. **a** Heatmap of Spearman’s rank correlations (*ρ*) between ion concentrations across groundwater samples from the ORFRC [16]. Color scale is the same as in **b**. Values above 0.75 are colored as if they were 0.75. **b** Heatmap of Spearman's rank correlations between ion concentrations and the relative abundances of the eight most abundant genera. **c** Concentrations of U and pH in groundwater samples from wells at the ORFRC. Symbols are colored red if the sample contains >5% *Rhodanobacter*. **d** Relative abundances of *Rhodanobacter* (red circles) and *Pseudomonas* (black x's) vs. concentrations of U and pH in groundwater samples from the ORFRC

We analyzed data from a field survey in which the concentrations of 30 inorganic ion parameters, including pH, were measured in groundwater from 93 wells at the ORFRC [16] (Supplemental Table S1). Many of these ions are known to be elevated in the contaminated wells and are associated with microbial community taxonomic shifts [14, 16]. In this dataset, we found that low pH is significantly correlated (Spearman’s rank correlation, false discovery rate <5%) with high concentrations of 19 inorganic ion parameters including U, Al, Mn, Ni, Co, Zn, Cd, Ca, NO_3_^−^, K, Pb, As, Cr, Mg, Be, Ga, Fe, SO_4_^2−^, and Cl^−^ (Fig. 1a, Supplemental Table S2).

We also analyzed 16S rDNA amplicon sequences that were collected alongside the geochemical data. We found that the relative abundance of *Rhodanobacter* is significantly positively correlated (false discovery rate <5%) with 15 inorganic ion parameters including low pH and high U, NO_3_^−^, Mn, Al, Co, Zn, Cd, Ni, Ca, Sr, K, Ba, SO_4_^2−^, and Cl^−^ (Fig. 1b, Table S2), while the other ions are not strongly correlated with *Rhodanobacter* (Fig. 1b, Table S2). We also examined correlations for the other seven genera that have the highest relative abundance on average in the field survey dataset. There are few strong positive correlations between high relative abundances of these other genera and high concentrations of any of the ions (Fig. 1b, Table S2). Rather, high relative abundances of the other genera are often negatively correlated with high concentrations of inorganic ions. This may indicate that several inorganic ions in the most contaminated wells limit the growth of these other taxa (Fig. 1b, Table S2) while permitting *Rhodanobacter* growth.

*Rhodanobacter* are occasionally observed in uncontaminated wells with a median relative abundance of 0.02%, but often dominate the microbial community in the most contaminated wells with low pH and high concentrations of U (Fig. 1c) and other inorganic ions (Figure S2). In one sample from well FW106, *Rhodanobacter* has a relative abundance of 78.5%. In fact, all of the wells with >5% *Rhodanobacter* are the most contaminated wells with both high U concentrations and low pH (Fig. 1c). While many *Rhodanobacter* isolates from the ORFRC grow more slowly than other isolates in laboratory culture media at low metal concentration, perhaps consistent with their low abundance in uncontaminated wells, there is evidence that *Rhodanobacter* isolates from the ORFRC are acid and metal tolerant [22, 26, 28]. Thus, we postulated that some, but not all, of the inorganic ions correlated with high relative abundance of *Rhodanobacter* are selective pressures that limit the growth of less resistant microbial taxa in the most contaminated wells.

### Identification of inorganic ions selectively inhibitory to a sensitive *Pseudomonas* isolate vs. a resistant *Rhodanobacter* isolate

We sought to identify which inorganic ions are selectively permissive for ion-resistant *Rhodanobacter* and restrict ion-sensitive taxa in the most contaminated wells. *Pseudomonas* have the sixth highest relative abundance of all genera in 16S rDNA amplicon data from the site (Fig. 1b, Table S1), but they are rarely abundant in the presence of high concentrations of inorganic ions and low pH (Fig. 1b, d; Figure S2). As an exemplar ion-sensitive isolate, we selected a *Pseudomonas* from an uncontaminated well (up-gradient from the contaminant plume), *Pseudomonas fluorescens* FW300-N2E2 (N2E2). We chose N2E2 because previous work indicated that this strain is relatively sensitive to some transition metals [23], and, as such, is likely representative of many ion-sensitive isolates from uncontaminated wells at the ORFRC.

**Fig. 2:**
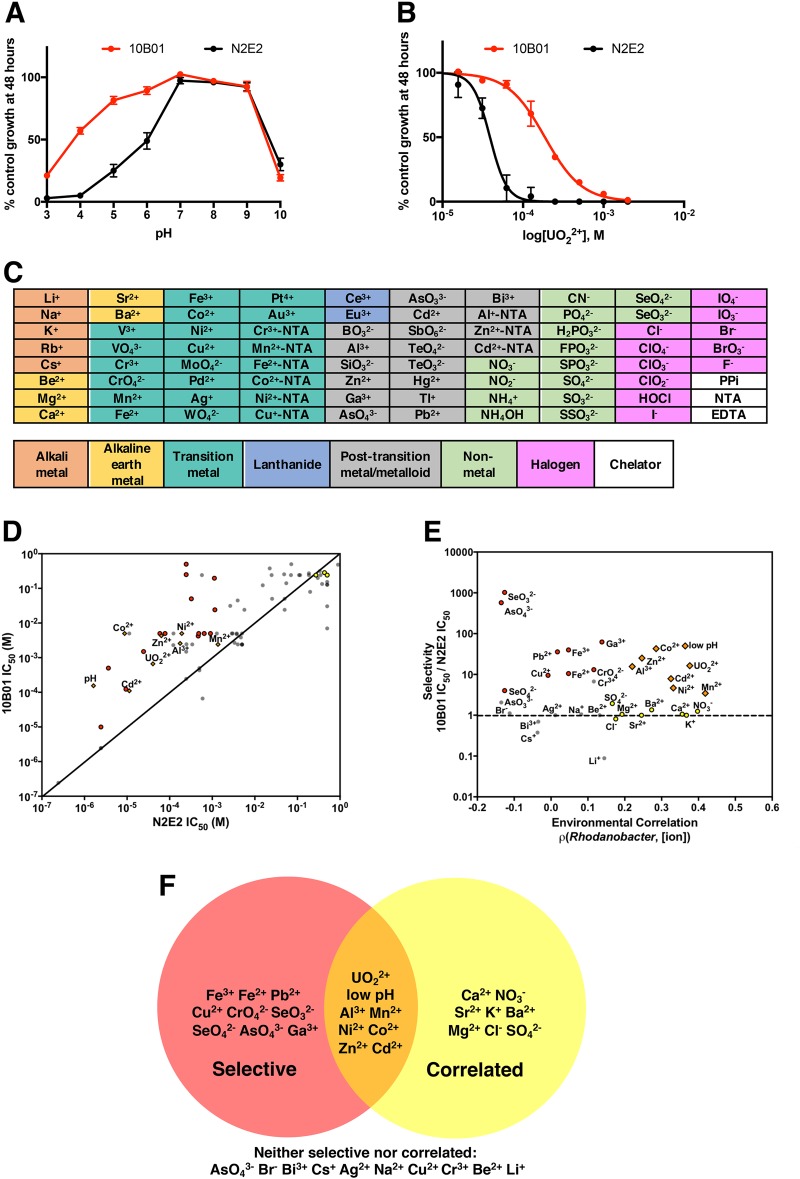
Comparisons of IC_50_s between a sensitive *Pseudomonas* isolate and a resistant *Rhodanobacter* isolate. **a** pH profiles for *Pseudomonas fluorescens* FW300-N2E2 (N2E2) and *Rhodanobacter* sp. FW104-10B01 (10B01). **b** Dose–response curves for inhibition of N2E2 and 10B01 by uranyl acetate (UO_2_^2+^). **c** The 80 inorganic ions used in dose–response assays. For some cations, nitrilotriacetic acid (NTA) complexes were prepared. Other abbreviations are PPi (pyrophosphate) and EDTA (ethylenediamine tetraacetic acid). **d** Comparisons of the IC_50_s for N2E2 and 10B01. pH is plotted as [H_3_O^+^]. The diagonal line represents equal IC_50_s for N2E2 and 10B01. **e** Comparisons of the N2E2/10B01 IC_50_ ratios (Selectivity) and the Spearman’s rank correlations between *Rhodanobacter* relative abundance and ion concentrations (Environmental Correlation). The horizontal line represents equal IC_50_s for N2E2 and 10B01. **f** Venn diagram showing the ions that are selectively more inhibitory of N2E2 than 10B01 (Selective, red), significantly positively correlated with increased *Rhodanobacter* relative abundance in the field survey (Correlated, yellow), or both (orange). Coloring in **d** and **e** is as in **f**

Because low pH and uranium are the major contaminants at the site, we measured the pH profile and quantified the inhibitory potency of U (uranyl acetate, U(VI), UO_2_^2+^) against a *Rhodanobacter* isolate from one of the most contaminated wells, *Rhodanobacter* sp. FW104-10B01 (10B01) and against N2E2. For the N2E2/10B01 comparisons, both strains were grown in R2A media aerobically. Consistent with these ions being selectivity determinants, we found that 10B01 is more resistant than N2E2 to low pH (Fig. 2a) and to UO_2_^2+^ (Fig. 2b).

To identify other inorganic ions to which 10B01 is resistant compared to N2E2, we determined the inhibitory potency of a panel of 80 inorganic ions (Fig. 2c) against both isolates grown in R2A media (Fig. 2d). We chose these ions because they likely represent the dominant oxidation states of most elements found in groundwater, or when multiple oxidation states are common, we included both. We quantify inhibitory potency as the concentration required to inhibit growth to 50% of uninhibited control cultures (IC_50_), and we quantify selectivity as the ratio between two IC_50_s (e.g., N2E2 IC_50_/10B01 IC_50_) (Supplemental Dataset, Table S5). We consider an ion “selective” if the 95% confidence intervals of the dose–response curves for the two organisms do not overlap and the IC_50_s differ by more than a factor of 2. By these criteria, alongside low pH and UO_2_^2+^, 10B01 is more resistant than N2E2 to 15 other selective ions representative of 13 elements measured in the field survey including Al^3+^, Mn^2+^, Ni^2+^, Co^2+^, Zn^2+^, Cd^2+^, Fe^2+^, Fe^3+^, Pb^2+^, Cu^2+^, CrO_4_^2−^, SeO_3_^2−^, SeO_4_^2−^, AsO_4_^3−^, and Ga^3+^ (Fig. 2d, e). While heavy metal resistance genes, such as cation metal efflux pumps, are present in *Rhodanobacter* genomes [22, 26, 28], similar resistance genes are present in the genomes of N2E2 and other *Pseudomonas* [23, 24]. While others have shown resistance of *Rhodanobacter* to low pH and high U [28], our results are the first systematic evaluation of which inorganic ions can select for *Rhodanobacter* similar to 10B01 over less-tolerant organisms from the ORFRC such as *P. fluorescens* FW300-N2E2.

Of the 17 selective ions to which 10B01 is resistant and for which we have measurements of the corresponding element in the field survey dataset, eight are positively correlated with *Rhodanobacter* relative abundance and are thus candidate-selective pressures that may favor *Rhodanobacter* over other taxa in the most contaminated wells at the ORFRC (Fig. 2e, f). These eight ions include low pH and high UO_2_^2+^, Mn^2+^, Al^3+^, Zn^2+^, Ni^2+^, Co^2+^, and Cd^2+^. All of these ions represent what is likely the dominant oxidation state of the element measured in the groundwater samples from the ORFRC. In contrast, N2E2 and 10B01 are similarly resistant to the alkali earth metal/alkali metal cations Ca^2+^, Sr^2+^, Ba^2+^, Mg^2+^, K^+^ and the anions Cl^−^, NO_3_^−^, and SO_4_^2−^, and thus these ions are less probable selectivity determinants. In general, these ions are less toxic compared to the selective ions (Supplemental Dataset Table S5). Finally, 10B01 is more resistant than N2E2 to the metal cations Fe^2+^, Fe^3+^, Pb^2+^, AsO_4_^3−^, and AsO_4_^3−^ and the oxyanions SeO_3_^2−^, SeO_4_^2−^, AsO_4_^3−^, and CrO_4_^2−^, but none of these ions are positively correlated with increased *Rhodanobacter* abundance. Although these ions could be selectivity determinants in some locations at the ORFRC, they do not seem to be dominant selective pressures in the most contaminated wells.

### Identification of inorganic ion toxicity thresholds in the field that can limit a sensitive *Pseudomonas* isolate and favor a resistant *Rhodanobacter* isolate

**Fig. 3:**
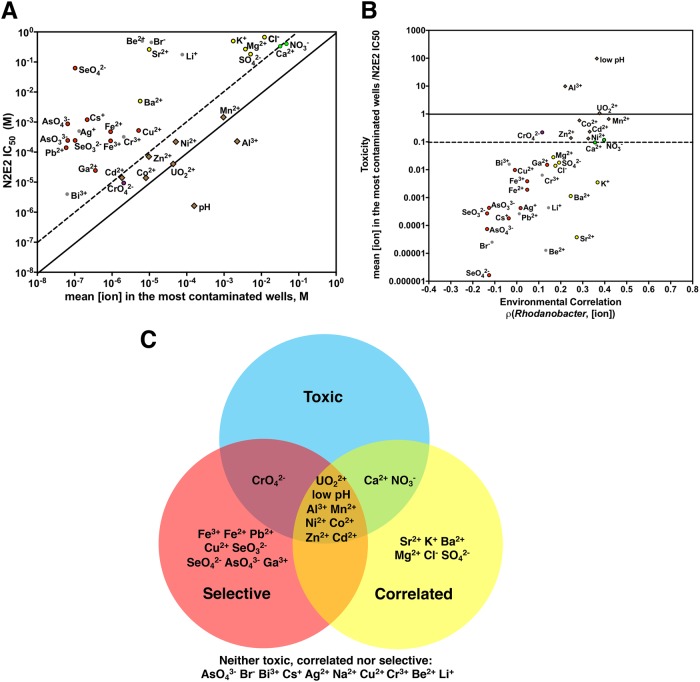
Comparisons of IC_50_s with field concentrations. **a** IC_50_s for *Pseudomonas fluorescens* FW300-N2E2 (N2E2) compared with the mean concentration of each ion in the most contaminated wells. pH is plotted as [H_3_O^+^]. Diagonal lines represent N2E2 IC_50_s equivalent to or 10-fold higher than the concentration in the most contaminated wells. **b** Comparisons between the ion toxicity in the most contaminated wells (ratio of mean [ion] in the most contaminated wells to the N2E2 IC_50_) and the environmental correlation (as defined in Fig. 2e). **c** Venn diagram representing the ions that are selectively inhibitory of N2E2 vs. 10B01 (Selective, red), significantly positively correlated with increased *Rhodanobacter* abundance in the field survey (Correlated, yellow) or reach toxic concentrations to N2E2 in the most contaminated wells (Toxic, blue). Coloring in **a** and **b** as in **c**

A selective ion must reach concentrations in the field that, while permissive for *Rhodanobacter*, limits the growth of other taxa. Thus, we compared the IC_50_s measured against N2E2 with the mean ion concentrations in the most contaminated wells (Fig. 3). We include wells in the set of “the most contaminated wells” if they have >5% *Rhodanobacter*. As noted above, all of the most contaminated wells have low pH and elevated concentrations of U (Fig. 1c) and other ions (Table S1).

The IC_50_s against N2E2 for the most toxic, selective ions including low pH, UO_2_^2+^, Mn^2+^, Al^3+^, NO_3_^−^, Ca^2+^, Cd^2+^, Co^2+^, and Zn^2+^ are within 10-fold of the mean concentrations of these ions in the most contaminated wells (Fig. 3a). We define all ions that meet this criterion as “toxic.” Strikingly, all of these toxic ions are significantly positively correlated with *Rhodanobacter* relative abundance in the field (Fig. 3b). Also, with the exception of Ca^2+^ and NO_3_^−^, all of these toxic ions are selectively inhibitory of N2E2 vs. 10B01 (Fig. 2). In general, there is good agreement between how strongly an ion is correlated with *Rhodanobacter* abundance and how toxic that ion is to N2E2 (Fig. 3b). For example, while Pb^2+^ and Al^3+^ have similar N2E2 IC_50_s (Fig. 3a), the Al concentration in the most contaminated wells is almost five orders of magnitude higher than the Pb concentration, and only Al is positively correlated with *Rhodanobacter* relative abundance. Thus, Al^3+^, but not Pb^2+^, is likely to be a selective ion at the ORFRC (Fig. 3a). On the other hand, Sr^2+^, K^+^, Ba^2+^, Mg^2+^, Cl^−^, and SO_4_^2^^−^ are positively correlated with increased *Rhodanobacter* relative abundance, but these ions are not selectively inhibitory of N2E2 vs. 10B01 (Figs. 2, 3c), and their concentrations in the contaminated wells are not toxic to N2E2 (Fig. 3b). Thus, it is likely that these ions are correlated with *Rhodanobacter* because they are solubilized by low pH, but they are not dominant selective pressures that impact the microbial community in the most contaminated wells.

The oxidation state of most ions was not measured in the field survey, but for Fe, Te, Se, Cr, and As, the toxicity varies depending on the element’s oxidation state (Figs. 2e, 3a). For example, while the CrO_4_^2−^ (Cr(VI)) IC_50_ is close to the field concentration of Cr, Cr^3+^ (Cr(III)) is much less toxic (Fig. 3c). Thus, although Cr is not strongly correlated with *Rhodanobacter* and is unlikely to be a dominant selective pressure across the ORFRC, it may be toxic to organisms like N2E2 in some of the most contaminated wells if Cr(VI) is the dominant oxidation state.

### The impact of varying growth conditions on inorganic ion toxicity

**Fig. 4:**
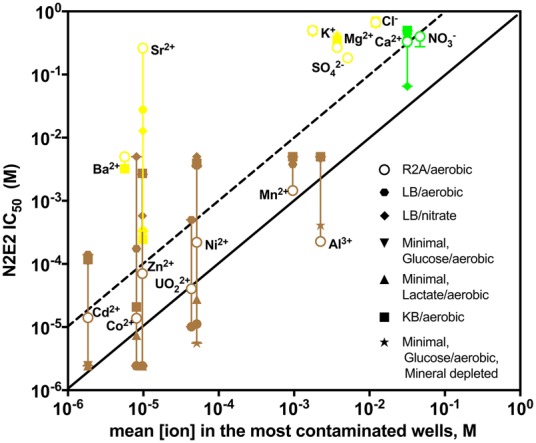
Impact of growth conditions on ion toxicity. IC_50_s for *Pseudomonas fluorescens* FW300-N2E2 (N2E2) compared with mean concentrations of each inorganic ion in the most contaminated wells. IC_50_s in each growth condition are indicated by symbols in the legend. Coloring is as in Fig. 3c. Diagonal lines represent N2E2 IC_50_s equivalent to and 10-fold above the mean [ion] in the most contaminated wells

Based on our initial aerobic growth assays in R2A medium, we identified low pH and high UO_2_^2+^, Mn^2+^, Al^3+^, Cd^2+^, Zn^2+^, Co^2+^, and Ni^2+^ as (a) correlated with *Rhodanobacter* relative abundance in the field (Fig. 1), (b) selectively inhibitory of N2E2 vs. 10B01 (Fig. 2), and (c) reaching concentrations toxic to N2E2 in the most contaminated wells (Fig. 3). However, conditions in the aquifer vary from the aerobic growth conditions in R2A. Thus, to systematically investigate condition-dependent ion sensitivity, we measured the impact on N2E2 IC_50_s of the carbon content in growth media, the carbon source, the terminal electron acceptor, the trace metal availability, and the iron availability (Fig. 4b). In Supplemental Note 1, we discuss the rationale for the growth conditions we chose and intriguing changes in N2E2 IC_50_s we observed. Here we primarily focus on the set of ions that are both correlated with *Rhodanobacter* relative abundance and reach toxic concentrations for N2E2 in the most contaminated wells (“Correlated” and “Toxic”, Fig. 3c). In particular, we sought to identify growth conditions in which UO_2_^2+^, Mn^2+^, Al^3+^, Cd^2+^, Co^2+^, Zn^2+^, Ni^2+^, Ca^2+^, or NO_3_^−^ are less toxic to N2E2 or Sr^2+^, Ba^2+^, Mg^2+^, Cl^−^, SO_4_^2−^, or K^+^ are more toxic. Large changes in IC_50_s for these ions might alter the list of ions that meet our criteria for a selective pressure that favors *Rhodanobacter* at the field site.

The results of the assays in different growth conditions do not alter our prediction of which ions are likely dominant selective pressures at the ORFRC (Fig. 4). The N2E2 IC_50_s for Mn^2+^, Al^3+^, Ca^2+^, NO_3_^−^, Ba^2+^, Mg^2+^, Cl^−^, SO_4_^2−^, and K^+^ are not greatly affected by changes in growth conditions (Fig. 4). In growth media with high concentrations of dissolved organic carbon (DOC) such as LB and KB, the toxicity of UO_2_^2+^, Zn^2+^, Co^2+^, Cd^2+^, and Ni^2+^ is alleviated because cations complex with organic acids to limit free metal activity [34]. However, ORFRC groundwater typically contains between 10 and 100 mg/L DOC [16, 35–38], which is several orders of magnitude lower than DOC in R2A (~3.6 g/L), LB (~20 g/L) or our minimal medium (~3.6 g/L). Thus, except in soils, where organic carbon can be up to 2% by weight [39], or when concentrated organic carbon is injected for uranium immobilization [14, 40], it is unlikely that DOC reaches sufficient levels to mitigate the toxicity of metals to sensitive microorganisms. Conversely, in minimal medium, at low carbon concentration, the N2E2 IC_50_ for Sr^2+^ is ~30-fold above the mean concentration in the most contaminated wells. Thus, it is possible that there are carbon-limited environments at the ORFRC where Sr^2+^ is inhibitory to sensitive microorganisms such as N2E2. However, Sr^2+^ is not selectively inhibitory of N2E2 vs. 10B01, and thus, it is unlikely that Sr^2+^ is an important selective pressure at the site.

Because 10B01 does not grow in our minimal medium, we could not evaluate as many growth conditions with 10B01 as with N2E2. However, comparison of the 10B01 IC_50_s from growth assays in R2A with mean ion concentrations in the most contaminated wells suggests that the free metal activity in this medium is close to the activity in ORFRC groundwater (Figure S3). For Al^3+^, Mn^2+^, Ca^2+^, and NO_3_^−^, the 10B01 IC_50_s are within a factor of 10 of the concentrations in the most contaminated wells. For UO_2_^2+^, Co^2+^, Zn^2+^, Ni^2+^, and Cd^2+^, the IC_50_s are within a factor of 100 of the concentrations in the contaminated wells. Because the most contaminated wells are permissive for 10B01 and other *Rhodanobacter*, it is unlikely that the free ion activity in the contaminated wells is more than 100-fold higher than in R2A.

Additionally, *Rhodanobacter* are often implicated in denitrification in the most contaminated wells [14, 21, 22, 27], and we were able to compare 10B01 IC_50_s between aerobic- and nitrate-reducing conditions (Table S5). As with N2E2, we observed some differences in ion toxicity to 10B01 depending on the terminal electron acceptor (Table S5), but not for UO_2_^2+^, Mn^2+^, Al^3+^, Co^2+^, Cd^2+^, Ni^2+^, and Zn^2+^. These results indicate that this set of ions are important selective pressures in the most contaminated wells regardless of whether nitrate reduction or aerobic respiration is the dominant terminal electron-accepting process.

### The sensitivity of panels of ORFRC isolates to ions at mean concentrations in the most contaminated wells

**Fig. 5:**
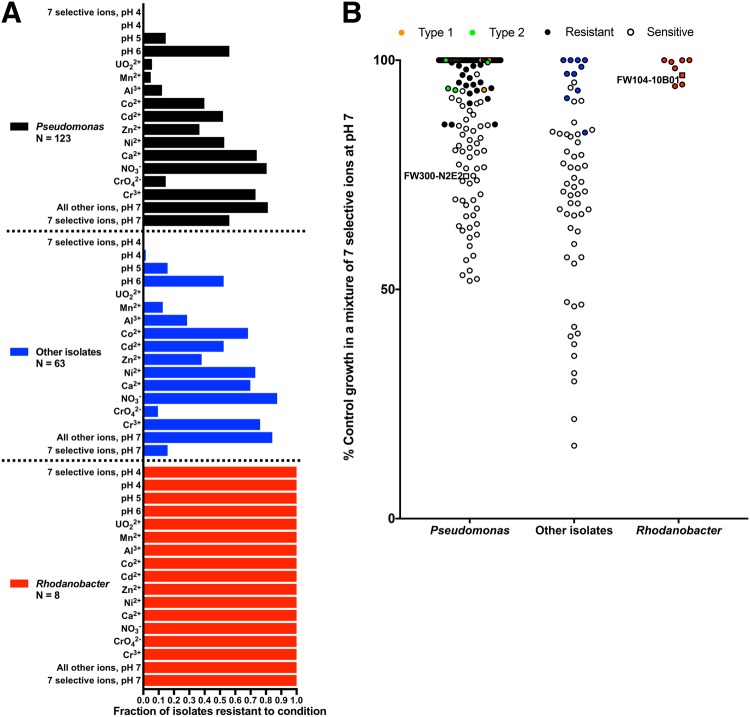
The sensitivity of panels of ORFRC isolates to ions at mean concentrations in the most contaminated wells. **a** The fraction of isolates that are resistant to mixtures of ions. Each ion is at its mean concentration in the most contaminated wells. Isolates are split into three categories: *Pseudomonas*, other isolates from uncontaminated wells, and *Rhodanobacter* isolates from contaminated wells. “7 selective ions” includes UO_2_^2+^, Mn^2+^, Al^3+^, Co^2+^, Cd^2+^, Zn^2+^, and Ni^2+^. “All other ions” are the 19 other ions we considered (Fig. 3c) aside from the seven selective ions, pH, Ca^2+^, NO_3_^−^, Cr^3+^, or CrO_4_^2−^. **b** Inhibition of isolates by a mixture of the seven selective ions at pH 7. Exact 16S rDNA V4 sequence matches with two dominant *Pseudomonas* sub-populations in a contaminated well, PTMW02, are colored orange (Type 1) and green (Type 2). % Control growth above 100% is plotted at 100%

To further test if low pH and high UO_2_^2+^, Mn^2+^, Al^3+^, Cd^2+^, Co^2+^, Zn^2+^, and Ni^2+^ are likely selective pressures that favor resistant microorganisms over sensitive microorganisms at our field site, we measured the growth of 194 bacterial isolates from the ORFRC, including N2E2 and 10B01, in the presence of a mixture of these eight ions at their mean concentrations in the most contaminated wells (Fig. 5). The mean concentrations of the inorganic ion parameters are reported in Table S1, and this mixture contains 4.5 μM UO_2_^2+^, 1 mM Mn^2+^, 2.25 mM Al^3+^, 2 μM Cd^2+^, 8 μM Co^2+^, 10 μM Zn^2+^, and 53 μM Ni^2+^ at pH 4 (Fig. 5a, Table S6). For these assays, we grew the isolates in R2A medium aerobically and quantified inhibition relative to control cultures grown in the absence of inhibitory ions (see Materials and methods and Table S6). Of the arrayed isolates, only eight *Rhodanobacter* from contaminated wells are capable of robust growth in all conditions we tested (Fig. 5a). None of the other 186 isolates, including 123 *Pseudomonas* and 63 other bacteria from uncontaminated wells were capable of growth in this condition.

As individual ions, few isolates are resistant to low pH (pH 4, 15%), UO_2_^2+^ (4%), Mn^2+^ (8%), or Al^3+^ (18%), but more isolates are resistant to Cd^2+^ (51%), Zn^2+^ (37%), Co^2+^ (49%), or Ni^2+^ (60%). Most isolates are resistant to the individual ions that met fewer of the criteria for a selective ion including 33 mM Ca^2+^ (73%), 4.8 mM NO_3_^−^ (83%), 2.15 μM Cr^3+^ (75%), or a mixture of all other 19 ions measured in the field survey at their mean concentrations in the most contaminated wells (72%) (Figs. 3c, 5a). In the mixture of all other ions, when multiple oxidation states represent an element measured in the field survey, we included both (e.g., we added 1 μM of both Fe^3+^ and Fe^2+^ for Fe). Although only 13% of isolates are resistant to 2.14 μM CrO_4_^2−^, Cr is not correlated with high *Rhodanobacter*, and thus, by our criteria, is unlikely to be a dominant selective pressure. However, we do not have data on whether Cr^3+^ or CrO_4_^2−^ is the major Cr species in the most contaminated wells. These results support our hypothesis that low pH, and high UO_2_^2+^, Mn^2+^, Al^3+^, Cd^2+^, Co^2+^, Zn^2+^, and Ni^2+^ are the likely dominant selective pressures in the contaminated wells at the ORFRC, while the other 22 ions we considered are probably less important.

We wished to classify the isolates from uncontaminated wells based on differential resistance to the dominant selective pressures in the most contaminated wells at the ORFRC. However, due to the extreme toxicity of the pH 4 mixture of all eight selective ions to all isolates aside from *Rhodanobacter*, we could not observe differential resistance phenotypes within the isolates from the uncontaminated wells. Thus, we measured resistance to a neutral pH mixture of UO_2_^2+^, Mn^2+^, Al^3+^, Cd^2+^, Co^2+^, Zn^2+^, and Ni^2+^ simulating the concentrations of these selective ions in the most contaminated wells (Fig. 5). One of the most contaminated wells, PTMW02, has a high relative abundance of *Pseudomonas*, despite concentrations of the eight selective ions close to the mean in the most contaminated wells (Table S1). Although the *Pseudomonas* isolates we tested are from uncontaminated wells, we found that all *Pseudomonas* isolates with 16S rDNA V4 regions identical to the two dominant sub-populations in PTMW02 are resistant to the ion mixture simulating the conditions in that well (Fig. 5b). PTMW02, with a pH of 4.78, has the highest pH of any of the most contaminated wells (Table S1), and many *Pseudomonas*, including the dominant populations in PTMW02, are capable of growth at pH 5. Together, these results help explain the success of resistant *Pseudomonas* strains in a contaminated well.

### Non-additive interactions between inorganic ions can influence toxicity thresholds

Inorganic ions can be synergistic or antagonistic with each other for inhibition of microorganisms [41]. We noticed that more isolates (42%) are resistant to the neutral pH mixture of UO_2_^2+^, Mn^2+^, Al^3+^, Cd^2+^, Co^2+^, Zn^2+^, and Ni^2+^ than to Mn^2+^ (8%) or UO_2_^2+^ (4%) alone (Fig. 5b). This suggests antagonism, and when we measured the N2E2 IC_50_s for UO_2_^2+^ in combination with Mn^2+^, we found the interaction to be antagonistic (Figure S6). Antagonism is known for interactions between UO_2_^2+^ and metals for inhibition of the aquatic plant, *Lemna aequinoctialis* [42] and for *Hydra viridissima* [43], but to our knowledge, this is the first report of such antagonism in bacteria. This antagonism does not alter our predictions of dominant selective pressures in the most contaminated wells, because combinations of Mn^2+^ with UO_2_^2+^ only alters the IC_50_s of these ions by less than a factor of 5 (Figure S6), and the mixture of the eight selective ions is much more inhibitory than all other 22 ions we considered (Fig. 5a). However, these non-additive interactions may be important in the complex gradients of multiple ions at the ORFRC.

## Discussion

In diverse environments, including the ORFRC, the increased prevalence of ion-resistant taxa is positively correlated with increased ion concentrations [5, 6, 16]. However, in complex ion gradients, the dominant selective ions are often difficult to identify. In this study, we developed a systematic, high-throughput approach for identifying selectively toxic inorganic ions. We found that low pH and high UO_2_^2+^, Mn^2+^, Al^3+^, Cd^2+^, Co^2+^, Zn^2+^, and Ni^2+^ are likely selective pressures in the most contaminated wells at the ORFRC because these ions are (a) positively correlated with *Rhodanobacter* relative abundance across the field site, (b) selectively inhibitory of non-*Rhodanobacter* isolates from uncontaminated wells, and (c) reach toxic concentrations in the contaminated wells that are inhibitory to most isolates from uncontaminated wells.

Our results point to specific areas where future field measurements will enhance our understanding of the ORFRC. For example, because the toxicity of some ions (e.g., UO_2_^2+^, Cd^2+^, Zn^2+^, Co^2+^, and Ni^2+^) varies as a function of organic carbon content, it is difficult to quantitatively rank the relative importance of each selective ion without more knowledge of the variable influence of natural organic matter on ion toxicity across the aquifer. In addition, our conceptual model of the ORFRC will be improved with more data on the prevalence and oxidation state of some ions. For example, Se, Te, As, Cr, and Fe vary dramatically in toxicity depending on oxidation state. More data on the redox speciation of these elements may enable the identification of locations in the aquifer where these ions are occasionally toxic to microbial populations. For some of the most toxic elements (e.g., Hg, Th, Pd, Ce, Cs, and Au), the N2E2 IC_50_ was below the lowest concentration we tested in our dose–response assays, but we have limited data on the prevalence of these ions. Mercury (Hg) has been measured in ORFRC groundwater at concentrations of up to 10 nM [44], which suggests that mercury concentrations could limit N2E2 growth at some locations in the field, and mercury efflux systems are observed in *Rhodanobacter* from the most contaminated wells at the ORFRC [45].

Many studies on the microbial ecology of metal-contaminated aquifers focus on the interpretation of taxonomic shifts in terms of probable physiological traits associated with UO_2_^2+^ bioreduction and immobilization [14, 25, 40, 46]. Future studies at the ORFRC should consider the impact of the toxic ions we identified on microbial metabolic capabilities implicated in controlling U mobility. For, example while *Rhodanobacter* are highly resistant to conditions in the contaminated wells, they are associated with nitrate reduction and U mobilization [14, 21, 25, 46]. Our results indicate that the conditions in the contaminated wells are permissive for *Rhodanobacter* independent of respiratory state, but other denitrifiers and the Fe^3+^- or SO_4_^2−^-reducing microorganisms that mediate U reduction [14, 25] may differ in sensitivity to the toxic ions in the contaminated wells. High-throughput dose–response assays can also be used to identify specific inhibitors of respiratory metabolisms, for example, to inhibit processes such as nitrate reduction that favor U oxidation or to stimulate processes such as U reduction [3, 30, 47]. Also, targeted interventions such as the addition of chelators [48], or choosing carbon sources and carbon concentrations to minimize metal toxicity [49] may improve the efficacy of U bioremediation efforts.

Our results suggest that future research should focus on understanding the mechanisms of resistance to the specific ions we identified as likely selective pressures at the ORFRC. These mechanisms of ion resistance could include, but are not limited to, biofilm formation [50], siderophore production, and metal efflux systems [24, 41, 50]. For example, metal efflux systems are common in both *Pseudomonas* [23, 24] and *Rhodanobacter* [21, 22, 27], but the ion specificity of the resistance mechanisms for UO_2_^2+^, Mn^2+^, Al^3+^, Cd^2+^, Co^2+^, Zn^2+^, and Ni^2+^ is poorly understood. Also, little is known about Mn^2+^ and Al^3+^ resistance in ORFRC isolates, but these ions are likely important selective pressures in the most contaminated wells. Finally, low pH, high NO_3_^−^ and high UO_2_^2+^ are commonly considered markers of contamination at the site and these ions are correlated with high *Rhodanobacter* abundance [14, 16, 17, 24, 25], but while UO_2_^2+^ and low pH are selective pressures due to toxicity, NO_3_^−^ is not. Thus, our results indicate that NO_3_^−^ is only likely to impact community composition in the most contaminated wells as an alternative terminal electron acceptor.

In the future, our approach can be applied to other environments with elevated concentrations of inorganic ions such as the rhizosphere [51], oil reservoirs [44], wastewater [10], or marine sediments [45]. While the ORFRC is an extreme example, there are indications that inorganic ion toxicity impacts microbial populations in many environments [5, 6, 52]. More broadly, understanding the biogeochemical controls on microbial activity in the environment is a central challenge of environmental microbiology, and we anticipate that further efforts to array biological and geochemical diversity in a format amenable to high-throughput cultivation will enable more rapid and accurate identification of the causal factors that impact microbial community composition and activity.

## Electronic supplementary material

Supplementary Materials

Supplementary Dataset with Tables S1-S6 is provided in a separate .xlsx file

## References

[1] Eisenhauer N, Schulz W, Scheu S, Jousset A (2012). Niche dimensionality links biodiversity and invasibility of microbial communities. Funct Ecol.

[2] Vellend M (2010). Conceptual synthesis in community ecology. Q Rev Biol.

[3] Carlson H, Deutschbauer A, Coates J (2017). Microbial metal resistance and metabolism across dynamic landscapes: high-throughput environmental microbiology. F1000Res.

[4] Atashgahi S, Sánchez-Andrea I, Heipieper HJ, van der Meer JR, Stams AJM, Smidt H (2018). Prospects for harnessing biocide resistance for bioremediation and detoxification. Science.

[5] Duxbury Trevor (1985). Ecological Aspects of Heavy Metal Responses in Microorganisms. Advances in Microbial Ecology.

[6] Gadd GM (2010). Metals, minerals and microbes: geomicrobiology and bioremediation. Microbiology.

[7] Tchounwou PB, Yedjou CG, Patlolla AK, Sutton DJ (2012). Heavy metal toxicity and the environment. EXS.

[8] Hinsinger P, Plassard C, Tang C, Jaillard B (2003). Origins of root-mediated pH changes in the rhizosphere and their responses to environmental constraints: a review. Plant Soil.

[9] Carson JK, Campbell L, Rooney D, Clipson N, Gleeson DB (2009). Minerals in soil select distinct bacterial communities in their microhabitats. FEMS Microbiol Ecol.

[10] Stepanauskas R, Glenn TC, Jagoe CH, Tuckfield RC, Lindell AH, McArthur JV (2005). Elevated microbial tolerance to metals and antibiotics in metal-contaminated industrial environments. Environ Sci Technol.

[11] Baker BJ, Banfield JF (2003). Microbial communities in acid mine drainage. FEMS Microbiol Ecol.

[12] Johnson DB, Hallberg KB (2003). The microbiology of acidic mine waters. Res Microbiol.

[13] Hug LA, Thomas BC, Brown CT, Frischkorn KR, Williams KH, Tringe SG (2015). Aquifer environment selects for microbial species cohorts in sediment and groundwater. ISME J.

[14] Li B, Wu WM, Watson DB, Cardenas E, Chao Y, Phillips DH (2018). Bacterial community shift and coexisting/coexcluding patterns revealed by network analysis in a uranium-contaminated site after bioreduction followed by reoxidation. Appl Environ Microbiol.

[15] Lin X, McKinley J, Resch CT, Kaluzny R, Lauber CL, Fredrickson J (2012). Spatial and temporal dynamics of the microbial community in the Hanford unconfined aquifer. ISME J.

[16] Smith MB, Rocha AM, Smillie CS, Olesen SW, Paradis C, Wu L (2015). Natural bacterial communities serve as quantitative geochemical biosensors. mBio.

[17] Brooks SC. Waste characteristics of the former S-3 ponds and outline of uranium chemistry relevant to NABIR Field Research Center Studies. 2001. 10.2172/814525.

[18] Gromet LP, Haskin LA, Korotev RL, Dymek RF (1984). The “North American shale composite”: its compilation, major and trace element characteristics. Geochim Cosmochim Acta.

[19] Reinhard C. T., Planavsky N. J., Robbins L. J., Partin C. A., Gill B. C., Lalonde S. V., Bekker A., Konhauser K. O., Lyons T. W. (2013). Proterozoic ocean redox and biogeochemical stasis. Proceedings of the National Academy of Sciences.

[20] Thorgersen MP, Lancaster WA, Vaccaro BJ, Poole FL, Rocha AM, Mehlhorn T (2015). Molybdenum availability is key to nitrate removal in contaminated groundwater environments. Appl Environ Microbiol.

[21] Hemme CL, Deng Y, Gentry TJ, Fields MW, Wu L, Barua S (2010). Metagenomic insights into evolution of a heavy metal-contaminated groundwater microbial community. ISME J.

[22] Hemme CL, Green SJ, Rishishwar L, Prakash O, Pettenato A, Chakraborty R (2016). Lateral gene transfer in a heavy metal-contaminated-groundwater microbial community. mBio.

[23] Price MN, Wetmore KM, Waters RJ, Callaghan M, Ray J, Liu H (2018). Mutant phenotypes for thousands of bacterial genes of unknown function. Nature.

[24] Thorgersen MP, Lancaster WA, Ge X, Zane GM, Wetmore KM, Vaccaro BJ (2017). Mechanisms of chromium and uranium toxicity in *Pseudomonas stutzeri* RCH2 grown under anaerobic nitrate-reducing conditions. Front Microbiol.

[25] Williams KH, Bargar JR, Lloyd JR, Lovley DR (2013). Bioremediation of uranium-contaminated groundwater: a systems approach to subsurface biogeochemistry. Curr Opin Biotechnol.

[26] Green SJ, Prakash O, Jasrotia P, Overholt WA, Cardenas E, Hubbard D (2012). Denitrifying bacteria from the genus *Rhodanobacter* dominate bacterial communities in the highly contaminated subsurface of a nuclear legacy waste site. Appl Environ Microbiol.

[27] Hemme CL, Tu Q, Shi Z, Qin Y, Gao W, Deng Y (2015). Comparative metagenomics reveals impact of contaminants on groundwater microbiomes. Front Microbiol.

[28] Prakash O, Green SJ, Jasrotia P, Overholt WA, Canion A, Watson DB (2012). *Rhodanobacter denitrificans* sp. nov., isolated from nitrate-rich zones of a contaminated aquifer. IJSEB.

[29] van den Heuvel RN, van der Biezen E, Jetten MSM, Hefting MM, Kartal B (2010). Denitrification at pH 4 by a soil-derived *Rhodanobacter*-dominated community. Environ Microbiol.

[30] Carlson HK, Stoeva MK, Justice NB, Sczesnak A, Mullan MR, Mosqueda LA (2015). Monofluorophosphate is a selective inhibitor of respiratory sulfate-reducing microorganisms. Environ Sci Technol.

[31] Ritz C, Baty F, Streibig JC, Gerhard D (2015). Dose–response analysis using R Xia Y (ed). PLoS ONE.

[32] Ritz C, Streibig JC (2005). Bioassay analysis using R. J Stat Softw.

[33] Wang Q, Garrity GM, Tiedje JM, Cole JR (2007). Naive Bayesian classifier for rapid assignment of rRNA sequences into the new bacterial taxonomy. Appl Environ Microbiol.

[34] Hughes MN, Poole RK (1991). Metal speciation and microbial growth—the hard (and soft) facts. J Gen Microbiol.

[35] Drewes JE, Fox P (1999). Fate of natural organic matter (NOM) during groundwater recharge using reclaimed water. Water Sci Technol.

[36] McCarthy JF, Williams TM, Liang LY, Jardine PM, Jolley LW, Taylor DL (1993). Mobility of natural organic-matter in a sandy aquifer. Environ Sci Technol.

[37] Wu X, Wu L, Liu Y, Zhang P, Li Q, Zhou J (2018). Microbial interactions with dissolved organic matter drive carbon dynamics and community succession. Front Microbiol.

[38] Davis JA (1984). Complexation of trace-metals by adsorbed natural organic-matter. Geochim Cosmochim Acta.

[39] Osman KT. Forest soils: properties and management. Springer International: Switzerland; 2013.

[40] Watson DB, Wu WM, Mehlhorn T, Tang G, Earles J, Lowe K (2013). In situ bioremediation of uranium with emulsified vegetable oil as the electron donor. Environ Sci Technol.

[41] Lemire JA, Harrison JJ, Turner RJ (2013). Antimicrobial activity of metals: mechanisms, molecular targets and applications. Nat Rev Microbiol.

[42] Charles AL, Markich SJ, Ralph P (2006). Toxicity of uranium and copper individually, and in combination, to a tropical freshwater macrophyte (*Lemna aequinoctialis*). Chemosphere.

[43] Hyne RV, Rippon GD, Ellender G (1992). pH-dependent uranium toxicity to freshwater hydra. Sci Total Environ.

[44] Pereira JSF, Moraes DP, Antes FG, Diehl LO, Santos MFP, Guimarães RCL (2010). Determination of metals and metalloids in light and heavy crude oil by ICP-MS after digestion by microwave-induced combustion. Microchem J.

[45] Hornberger MI, Luoma SN, van Geen A, Fuller C, Anima R (1999). Historical trends of metals in the sediments of San Francisco Bay, California. Mar Chem.

[46] Fields MW, Yan T, Rhee SK, Carroll SL, Jardine PM, Watson DB (2005). Impacts on microbial communities and cultivable isolates from groundwater contaminated with high levels of nitric acid-uranium waste. FEMS Microbiol Ecol.

[47] Carlson HK, Mullan MR, Mosqueda LA, Chen S, Arkin MR, Coates JD (2017). High-throughput screening to identify potent and specific inhibitors of microbial sulfate reduction. Environ Sci Technol.

[48] Leštan D, Luo CL, Li XD (2008). The use of chelating agents in the remediation of metal-contaminated soils: a review. Environ Pollut.

[49] Vanengelen MR, Field EK, Gerlach R, Lee BD, Apel WA, Peyton BM (2010). UO(2) 2+ speciation determines uranium toxicity and bioaccumulation in an environmental *Pseudomonas* sp. isolate. Environ Toxicol Chem.

[50] Workentine ML, Harrison JJ, Stenroos PU, Ceri H, Turner RJ (2008). *Pseudomonas fluorescens'* view of the periodic table. Environ Microbiol.

[51] Giller KE, Witter E, Mcgrath SP (1998). Toxicity of heavy metals to microorganisms and microbial processes in agricultural soils: a review. Soil Biol Biochem.

[52] Fierer Noah (2017). Embracing the unknown: disentangling the complexities of the soil microbiome. Nature Reviews Microbiology.

