# Supplementary material for: The selective pressures on the microbial community in a metal-contaminated aquifer
Source: ISME J. 2018 Dec 6;13(4):937–49. doi: 10.1038/s41396-018-0328-1 (PMC6461962; doi:10.1038/s41396-018-0328-1)
Supplement: Supplementary file 1 — Supplementary Materials [file 41396_2018_328_MOESM1_ESM.docx]

**Supplementary Materials**

**The selective pressures on the microbial community in a metal-contaminated aquifer**

**Running Title:** Selective inorganic ions in a contaminated aquifer

Hans K. Carlson^1,*^, Morgan N. Price^1^, Mark Callaghan^1^, Alex Aaring^2^, Romy Chakraborty^2^, Hualan Liu^1^, Adam P. Arkin^1,3^, Adam M. Deutschbauer^1,*^

Addresses: ^1^Environmental Genomics and Systems Biology Division, Lawrence Berkeley National Laboratory, Berkeley, CA 94720, USA; ^2^Earth and Environmental Sciences Area, Lawrence Berkeley National Laboratory, Berkeley, CA 94720, USA; ^3^Department of Bioengineering, University of California, Berkeley, CA 94720, USA

* To whom correspondence should be addressed:

Hans K. Carlson: [HKCarlson@lbl.gov](mailto:HKCarlson@lbl.gov)

Adam M. Deutschbauer: AMDeutschbauer@lbl.gov

**Supplementary Materials contains Supplementary Methods, Supplementary Note, Supplementary Figures S1-S6, and a Supplementary Dataset with Supplementary Tables S1-S6 is provided as a separate .xlsx file.**

**Supplementary Dataset with Tables S1-S6 is provided in a separate .xlsx file**

**Table S1.** **Field Survey Data** 16S amplicon sequencing data and concentrations of various geochemical parameters identified as possible controls on N2E2 based on our study. Data from Smith, M. B. *et al.* Natural Bacterial Communities Serve as Quantitative Geochemical Biosensors. *mBio* **6,** e00326–15–13 (2015) and personal communication with the authors.

**Table S2.** **Field Correlations.** Spearman correlations between relative abundances of the *Pseudomonas* and *Rhodanobacter* genera from 16S amplicon sequencing data and concentrations of various geochemical parameters identified as possible controls on N2E2 based on our study. Data from Smith, M. B. *et al.* Natural Bacterial Communities Serve as Quantitative Geochemical Biosensors. *mBio* **6,** e00326–15–13 (2015) and personal communication with the authors.

**Table S3. 80 Inorganic Ion Array.** 80 inorganic ion array 96-well plate layout. Compound name, CAS numbers, and stock concentrations in M and mM are indicated.

**Table S4. Media Recipes.** Media recipes for King's B (KB) and chemically defined minimal medium (minimal medium) used for growth assays.

**Table S5.** **IC_50_s.** IC_50_ concentrations (M) and selectivity indices (SI) for 80 inorganic ions against *Rhodanobacter sp.* FW104-10B01 and *Pseudomonas fluorescens* FW300-N2E2 in different growth conditions.

**Table S6.** **Arrayed Isolates.** Arrayed ORFRC isolate sensitivity to individual components and mixtures of compounds at the mean concentrations in groundwater samples with >5% *Rhodanobacter*. % inhibition relative to controls is reported, and significant inhibition is indicated for cultures inhibited more than 2 standard deviations below the mean of positive control no stress cultures.

**Supplementary Methods**

**Quantification of synergistic inhibition**

Synergistic inhibition between inhibitors was assessed using the equation for Fractional Inhibitory Concentration Index (FICI) based on the IC_50_ for each inhibitor A and B in the absence (IC­_50_(A), IC_50_(B)) or presence of the other inhibitor (IC­_50_(AB), IC_50_(BA)):

FICI = FIC_A_ + FIC_B_ = IC_50_(AB)/IC_50_(A) + IC_50_(BA)/IC_50_(B).

A FICI score < 0.5 implies synergism, whereas a FICI score > 2 implies antagonism. An FICI between 1 and 2 implies indifference, and by convention, the greatest absolute value FICI score is reported (EUCAST, 2000).

European Committee for Antimicrobial Susceptibility Testing of the European Society of Clinical M, Infectious D (2000). EUCAST Definitive Document E.Def 1.2, May 2000: Terminology relating to methods for the determination of susceptibility of bacteria to antimicrobial agents. *Clinical microbiology and infection : the official publication of the European Society of Clinical Microbiology and Infectious Diseases* **6:** 503-508.

**Supplementary Note 1**

**Insights into variability and mechanisms of inorganic ion resistance from dose-response assays with *P. fluorescens* FW300-N2E2**

Because metal toxicity is controlled by free metal activity (Nowack and VanBriesen, 2005; Leštan *et al.*, 2008; Hughes and Poole, 1991) we measured the impact of varying organic carbon content on ion toxicity. We also assessed how different metabolic states of bacterial cells are differentially susceptible to antimetabolic ions. For example, the enzymes of different carbon utilization pathways are differentially susceptible to inhibitory ions (Ong *et al.*, 2015; Klemperer, 1950; Benabe *et al.*, 1987). Under aerobic conditions, toxic reactive oxygen species are generated by redox active metals (Lemire *et al.*, 2013; Strlič *et al.*, 2003), while under nitrate-reducing conditions, various oxyanion substrates of nitrate reductases are differentially toxic depending on whether they are detoxified or converted to a more toxic form by the enzyme (Oremland and Stolz, 2000; Oremland and Capone, 1988; Garbisu *et al.*, 1996). Under trace metal depleted conditions, toxic ions can compete with nutritional metals for uptake systems (Lemire *et al.*, 2013; Braud *et al.*, 2009; Wichard *et al.*, 2009; Teitzel and Parsek, 2003; Enoch and Lester, 1972).

**Organic carbon content**

To evaluate how N2E2 IC_50_s are impacted by organic carbon content we measured the inhibitory potency of the 80 inorganic compounds against N2E2 grown in a chemically-defined minimal medium containing ~6g/L organic carbon and in a rich media (LB, Luria-Bertani broth) with ~20 g/L organic carbon (Figure S4B). We observed lower N2E2 IC­_50_s in minimal medium relative to rich medium for UO_2_^2+^ and the transition metals Ni^2+^, Cd^2+^, Zn^2+^ and Co^2+^ such that these parameters are likely not inhibitory to sensitive, background organisms such as N2E2 in carbon rich environments such as organic rich soil horizons (Osman, 2013) or under carbon injection regimes at the ORFRC (Li *et al.*, 2018; Watson *et al.*, 2013), but will be toxic in typical low carbon aquifer environments with measured dissolved organic carbon between 10 and 100 mg/L (McCarthy *et al.*, 1993; Drewes and Fox, 1999; Davis, 1984; Smith *et al.*, 2015). In some cases, rich/minimal IC_50_ ratios are greater than 10^3^, but nitrilotriacetic acid (NTA) complexation alleviated ion toxicity in minimal media (Figure S5, Supplementary Dataset, Table S5). Together, these results are consistent with the view that the ion binding capacity of growth media controls toxicity (Nowack and VanBriesen, 2005; Leštan *et al.*, 2008; Hughes and Poole, 1991).

Similar to the impact of the organic carbon content in growth media, the capacity to form biofilms is likely to give some microorganisms an advantage in the contaminated wells. Biofilm formation by *Pseudomonas* can increase the IC_50_s of inorganic ions by over an order of magnitude, likely due to the ion binding capacity of the extracellular polysaccharide matrix produced by sessile cells (Workentine *et al.*, 2008). However, in no known case does biofilm formation increase the sensitivity of a strain to inorganic ions (Workentine *et al.*, 2008).

**Organic carbon source**

We evaluated how changing the carbon source from glucose to lactate impacts inhibitory potency (Figure S4C). In general, we saw very minor influence of lactate on IC_50_s, aside from a few compounds that may be antimetabolites for the enzymes involved in carbon catabolism. The enzymes of different catabolic pathways have different susceptibilities to inorganic compounds (Ong *et al.*, 2015) . VO_4_^3-^ and Be^2+^ are more inhibitory of glucose grown N2E2 cultures than lactate grown cultures. Both compounds can interfere with phosphate transfer reactions in glycolysis (Benabe *et al.*, 1987; Klemperer, 1950). We did not observe any compounds with greater inhibitory potency under lactate utilizing conditions.

**Terminal electron acceptor**

N2E2, like many Pseudomonads, is capable of complete denitrification of NO_3_^-^ to N_2_ (Thorgersen *et al.*, 2015b). We determined the IC_50_s of the 80 inorganic compounds on the anaerobic growth of N2E2 with LB as the carbon source and electron donor and 10 mM nitrate as the sole electron acceptor (Figure S4D). In general, the redox state for Te, Se, As and Cr has a strong impact on toxicity of these elements, making them differentially inhibitory of nitrate-reducing versus aerobic cultures (Supplementary Dataset, Table S6). Several inorganic substrates of nitrate reductase are more inhibitory of aerobic cultures including AsO_4_^3-^, AsO_3_^3-^, CrO_4_^2-^, SeO_3_^2-^ and TeO_3_^2-^. Reduction of these compounds to less toxic products by *Pseudomonas* and other bacteria is known (Oremland and Stolz, 2000; Garbisu *et al.*, 1996). In contrast, TeO_4_^2-^, IO_3_^-^, IO_4_^-^ and BrO_3_^-^ are more inhibitory of nitrate-reducing cultures likely because they are reduced to more toxic compounds by nitrate-reductases. Because, Se, Te, Cr and As oxyanions can vary widely in their inhibitory potency depending on redox state of the element and whether N2E2 is respiring oxygen or nitrate, we suggest that these compounds should also be considered alongside molybdate limitation (Thorgersen *et al.*, 2015b) in future studies as possible constraints on growth and nitrate reduction in the most contaminated wells.

Co^2+^ is more inhibitory of aerobic cultures and Zn^2+^ is more inhibitory of nitrate-reducing cultures. The mechanisms of metal toxicity can be complex, but aerobically redox stress and the formation of reactive oxygen species is important (Lemire *et al.*, 2013). Co^2+^ is a better catalyst of Fenton chemistry than Zn^2+^ (Strlič *et al.*, 2003), while under nitrate-reducing conditions, Zn may interfere with Cu uptake and cofactor formation in the nitrite reductase NirK, or the nitrous oxide reductase Nos (Kraft *et al.*, 2011).

**Iron and trace mineral depletion**

Nutritional metals supply essential metallocofactors, and trace metal limitation can have a dramatic effect on microbial activity (Lemire *et al.*, 2013; Braud *et al.*, 2009). Thus, we measured the inhibitory potency of the 80 inorganic ions in an iron-limited rich media widely used in *Pseudomonas* studies, King’s B broth (KB) (Duffy and Défago, 1999) (Figure S4E) and in a minimal medium depleted in trace minerals (Figure S2F). Co^2+^, Sr^2+^, and VO_4_^2-^ are more inhibitory of N2E2 growth in KB versus LB and are known to interfere with iron uptake mechanisms (Figure S4) (Braud *et al.*, 2009). Ga^3+^, another iron analog (Lemire *et al.*, 2013) was slightly more inhibitory of KB cultures. The carbon sources in LB are simple sugars, amino acids and peptides whereas in KB they are glycerol and amino acids. As with our comparison of lactate and glucose as a carbon source, Be^2+^ is more inhibitory of cultures relying on substrate-level phosphorylation (e.g. LB, glucose defined) (Klemperer, 1950). VO_4_^3-^ is more inhibitory of KB cultures and in this context it may be both an iron analog (Wichard *et al.*, 2009; Braud *et al.*, 2009) as well as a phosphate analog (Benabe *et al.*, 1987).

Several compounds are more inhibitory of trace mineral depleted cultures (Figure S2F). Ni^2+^, Al^3+^, and Pb^2+^ interfere with transition metal uptake (Teitzel and Parsek, 2003; Braud *et al.*, 2009) while TeO_4_^2-^ and WO_4_^2-^  can interfere with molybdate or sulfate uptake both of which are depleted in these conditions (Wichard *et al.*, 2009; Enoch and Lester, 1972; Prins *et al.*, 1980). CN^-^ is nearly 3 orders of magnitude more inhibitory of mineral replete cultures. CN^-^ binds to respiratory metallocofactors, but its degradation is catalyzed by metal siderophore complexes under metal depleted conditions by *Pseudomonas* (Luque-Almagro *et al.*, 2005; Chen and Kunz, 1997).

Further work will evaluate how trace nutrient depletion impacts microbial communities at the ORFRC. For example, molybdenum limitation may limit nitrate reduction activity in some areas of the ORFRC and select for organisms with higher molydenum affinity (Thorgersen *et al.*, 2015a). By extending the approach we used in this study, a high-throughput dose-response strategy can be used to both determine nutrient limitation thresholds for nitrogen, phosphate, sulfur and trace vitamins/minerals and systematically determine toxicity thresholds for specific inhibitors of nutrient uptake systems (Carlson *et al.*, 2017).

**Supplementary References**

Benabe JE, Echegoyen LA, Pastrana B, Martínez-Maldonado M. (1987). Mechanism of inhibition of glycolysis by vanadate. *J Biol Chem* **262**: 9555–9560.

Braud A, Hoegy F, Jezequel K, Lebeau T, Schalk IJ. (2009). New insights into the metal specificity of the *Pseudomonas aeruginosa* pyoverdine-iron uptake pathway. *Environ Microbiol* **11**: 1079–1091.

Carlson H, Deutschbauer A, Coates J. (2017). Microbial metal resistance and metabolism across dynamic landscapes: high-throughput environmental microbiology. *F1000Res* **6**: 1026–8.

Chen JL, Kunz DA. (1997). Cyanide utilization in Pseudomonas fluorescens NCIMB 11764 involves a putative siderophore. *FEMS Microbiology Letters* **156**: 61–67.

Davis JA. (1984). Complexation of Trace-Metals by Adsorbed Natural Organic-Matter. *Geochimica et Cosmochimica Acta* **48**: 679–691.

Drewes JE, Fox P. (1999). Fate of natural organic matter (NOM) during groundwater recharge using reclaimed water. *Water Science and Technology* **40**: 241–248.

Duffy BK, Défago G. (1999). Environmental factors modulating antibiotic and siderophore biosynthesis by *Pseudomonas fluorescens* biocontrol strains. *Applied and Environmental Microbiology* **65**: 2429–2438.

Enoch HG, Lester RL. (1972). Effects of molybdate, tungstate, and selenium compounds on formate dehydrogenase and other enzyme systems in *Escherichia coli.* *Journal of Bacteriology* **110**: 1032–1040.

Garbisu C, Ishii T, Leighton T, Buchanan BB. (1996). Bacterial reduction of selenite to elemental selenium. *Chemical Geology* **132**: 199–204.

Klemperer FW. (1950). The effect of beryllium on certain enzymes. *J Biol Chem* **187**: 189–196.

Kraft B, Strous M, Tegetmeyer HE. (2011). Microbial nitrate respiration: Genes, enzymes and environmental distribution. *Journal of Biotechnology* **155**: 104–117.

Lemire JA, Harrison JJ, Turner RJ. (2013). Antimicrobial activity of metals: mechanisms, molecular targets and applications. *Nat Rev Micro* **11**: 371–384.

Li B, Wu W-M, Watson DB, Cardenas E, Chao Y, Phillips DH, *et al.* (2018). Bacterial Community Shift and Coexisting/Coexcluding Patterns Revealed by Network Analysis in a Uranium-Contaminated Site after Bioreduction Followed by Reoxidation. Nojiri H (ed). *Applied and Environmental Microbiology* **84**: e02885–17.

Luque-Almagro VM, Huertas MJ, Martinez-Luque M, Moreno-Vivian C, Roldan MD, Garcia-Gil LJ, *et al.* (2005). Bacterial Degradation of Cyanide and Its Metal Complexes under Alkaline Conditions. *Applied and Environmental Microbiology* **71**: 940–947.

McCarthy JF, Williams TM, Liang LY, Jardine PM, Jolley LW, Taylor DL, *et al.* (1993). Mobility of Natural Organic-Matter in a Sandy Aquifer. *Environ Sci Technol* **27**: 667–676.

Ong C-LY, Walker MJ, McEwan AG. (2015). Zinc disrupts central carbon metabolism and capsule biosynthesis in *Streptococcus pyogenes*. *Nature Publishing Group* 1–10.

Oremland RS, Stolz J. (2000). Dissimilatory Reduction of Selenate and Arsenate in Nature. In: *Environmental Microbe-Metal Interactions*. American Society of Microbiology, pp 199–224.

Osman KT. (2013). Forest soils: properties and management.

Prins RA, Cliné-Theil W, Malestein A, Counotte GH. (1980). Inhibition of nitrate reduction in some rumen bacteria by tungstate. *Applied and Environmental Microbiology* **40**: 163–165.

Smith MB, Rocha AM, Smillie CS, Olesen SW, Paradis C, Wu L, *et al.* (2015). Natural Bacterial Communities Serve as Quantitative Geochemical Biosensors. *mBio* **6**: e00326–15–13.

Strlič M, Kolar J, Šelih VS, Kočar D, Pihlar B. (2003). A comparative study of several transition metals in Fenton-like reaction systems at circum-neutral pH. *Acta Chimica Slovenica* **50**: 619–632.

Teitzel GM, Parsek MR. (2003). Heavy metal resistance of biofilm and planktonic Pseudomonas aeruginosa. *Applied and Environmental Microbiology* **69**: 2313–2320.

Thorgersen MP, Lancaster WA, Vaccaro BJ, Poole FL, Rocha AM, Mehlhorn T, *et al.* (2015a). Molybdenum Availability Is Key to Nitrate Removal in Contaminated Groundwater Environments. *Applied and Environmental Microbiology* **81**: 4976–4983.

Thorgersen MP, Lancaster WA, Vaccaro BJ, Poole FL, Rocha AM, Mehlhorn T, *et al.* (2015b). Molybdenum Availability Is Key to Nitrate Removal in Contaminated Groundwater Environments Voordouw G (ed). *Applied and Environmental Microbiology* **81**: 4976–4983.

Watson DB, Wu W-M, Mehlhorn T, Tang G, Earles J, Lowe K, *et al.* (2013). In situ bioremediation of uranium with emulsified vegetable oil as the electron donor. *Environ Sci Technol* **47**: 6440–6448.

Wichard T, Bellenger J-P, Morel FMM, Kraepiel AML. (2009). Role of the Siderophore Azotobactin in the Bacterial Acquisition of Nitrogenase Metal Cofactors. *Environ Sci Technol* **43**: 7218–7224.

Workentine ML, Harrison JJ, Stenroos PU, Ceri H, Turner RJ. (2008). *Pseudomonas fluorescens'* view of the periodic table. *Environ Microbiol* **10**: 238–250.

**Supplementary Figures**

**Figure S1.** IC_50_s measured in replicate rich media (LB) aerobic dose-response assays conducted on different days. All compounds have overlapping 95% confidence intervals (Materials and Methods).

**Figure S2.** Relative abundances of *Rhodanobacter* (red circles) and *Pseudomonas* (black x's) versus concentrations for the geochemical parameters that are most correlated with *Rhodanobacter*.

**Figure S3.** IC_50_s for *Rhodanobacter sp.* FW104-10B01 (10B01) compared with mean concentrations of inorganic ions in the most contaminated wells. pH is represented by [H_3_O^+^]. Diagonal lines represent N2E2 IC_50_s equivalent to, 10-fold above and 100-fold above the mean [ion] in the most contaminated wells.

**Figure S4.** Influence of growth conditions on inhibitory potencies. Selected ions with altered inhibitory potency are labelled. **A.** IC_50_s for ions against N2E2 and 10B01 grown in R2A medium. **B.** IC_50_s for ions against N2E2 grown in minimal medium (with glucose as the sole carbon source) or rich medium (LB). **C.** IC_50_s for ions against N2E2 grown in minimal media with lactate or glucose as a carbon source. **D.** IC_50_s for ions against N2E2 grown in LB aerobically or anaerobically with nitrate as the sole electron acceptor. **E.** IC_50_s for ions against N2E2 grown in minimal media in mineral depleted conditions (0.25x trace mineral stock) or mineral replete conditions. **F.** IC_50_s for ions against N2E2 grown in iron-limited King’s B media (KB) or iron-replete LB. **G.** The fold change in N2E2 IC_50_s is plotted for various comparisons. Coloring is as in Figure 3C in the main text.


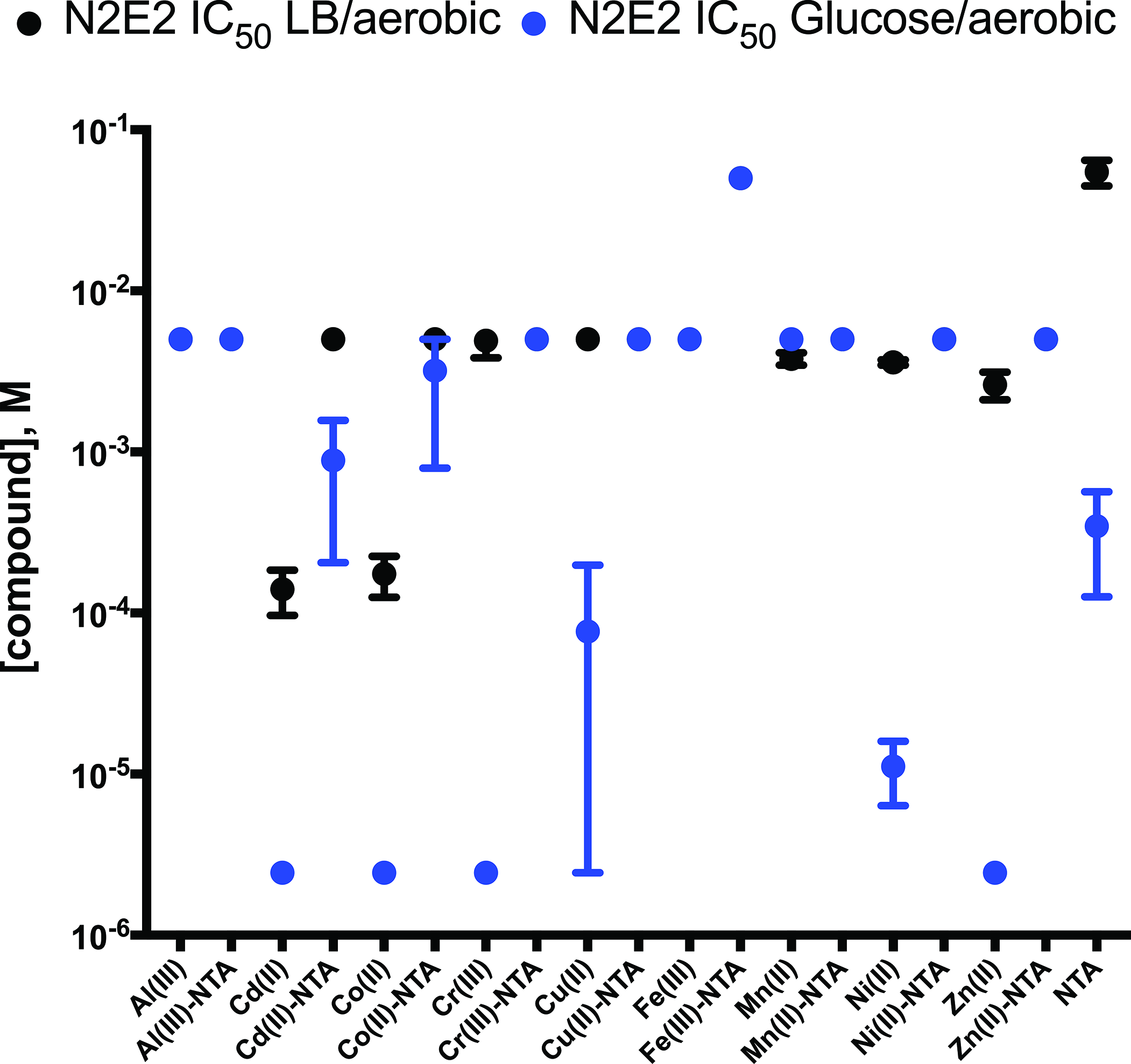


**Figure S5.** Influence of chelation on transition metal inhibitory potency. Inhibitory potencies (IC_50_, 95% CI) of selected free ions or NTA complexes against *Pseudomonas fluorescens* FW300-N2E2 grown aerobically in either rich media (LB/aerobic) or minimal media (Glucose/aerobic).

**Figure S6.** Inhibition of *Pseudomonas fluorescens* FW300-N2E2 (N2E2) for combinations of Mn and U. The fractional inhibition concentration index (FICI, Supplementary Materials and Methods) is reported and indicates antagonism.
